# Prevalence of postural musculoskeletal symptoms among dental students in United Arab Emirates

**DOI:** 10.1186/s12891-020-03887-x

**Published:** 2021-01-06

**Authors:** Raghad Hashim, Afraa Salah, Fatemeh Mayahi, Saeedeh Haidary

**Affiliations:** Growth and Development Department, Ajman University, Emirate of Ajman, P.O. Box 346, Ajman, United Arab Emirates

**Keywords:** Dental students, Musculoskeletal pain, Occupational health

## Abstract

**Background:**

The purpose of the present study was to determine the prevalence of neck, shoulder, and low-back pain and to examine factors associated with musculoskeletal pain (MSP) among dental students at Ajman University and Ras Al Khaimah College of Dental Sciences in United Arab Emirates (UAE).

**Method:**

A cross-sectional study was conducted among dental students using an online questionnaire, which was a modified version of the Standardized Nordic questionnaire, focused on neck, shoulder and low-back pain in the past week and the past year.

**Results:**

A total of two hundred and two dental students (out of 368) responded to the questionnaire. The majority were female 75.2%. The prevalence of MSP in at least one body site in the past week, and in the past year was 48.5 and 68.3% respectively. The factors significantly associated with MSP in at least one body site at any time were having history of trauma (*P* = 0.009), lack of exercise (*P* = 0.001), longer clinical sessions (*P* = 0.000), and higher BMI (*P* = 0.010).

**Conclusion:**

The present study indicates that the prevalence of MSP among dental students in the UAE is high. Careful attention from dental colleges is needed to increase students’ awareness of this problem. This study contributed to better understanding of MSP among dental professionals.

**Supplementary Information:**

The online version contains supplementary material available at 10.1186/s12891-020-03887-x.

## Background

The musculoskeletal health of dental professionals has been the subject of numerous studies world-wide, and their focus has been on the pain experienced by the practitioner [1]. A recent review conducted by Moodley and colleagues [2] demonstrated that the most common sites for musculoskeletal pain are the neck, lower-back and shoulders. The prevalence of dentists’ discomfort in the neck, shoulders, and lower-back as suggested in many studies conducted around the world ranged from 64 to 93% [3]. Thus, work related musculoskeletal disorder (MSD) is a common occupational hazard among dentists.

The nature of dental work, which includes repetitive movement, long work sessions and awkward static postures, contributes to musculoskeletal problems amongst dentists [1]. The costs of these problems are substantial, both in terms of money and in terms of work time lost [4]; these musculoskeletal problems may result in lower work productivity, have an adverse effect on the quality of work and efficiency, lead to a shortened working lifespan, and contribute to occupational disability, which constitute a major health challenge for individual and healthcare system around the world [5, 6]. A study conducted by Oberg [7] found that the loss of income due to musculoskeletal pain in the dentistry profession is greater than $40 million. Long term MSDs could affect one’s quality of life. Therefore, identifying factors associated with musculoskeletal problems in the dental workplace is of high importance [8].

Various studies have been performed to record self-perceived health and health-related behaviors of dental students in other countries [9, 10], but little is known about the health of dental students in the United Arab Emirates (UAE). Currently, only one study, conducted by Al-Ali and Hashim [11], has investigated occupational health problems among dentists in the UAE; this study demonstrated that musculoskeletal disorders were the most common (68%) occupational health problem.

This study aimed to determine the prevalence and nature of occupational-related musculoskeletal pain (MSP) and to explore factors associated with MSP among undergraduate dental students in two main dental colleges in the United Arab Emirates.

## Methods

A cross-sectional study was conducted among undergraduate dental students in two main dental colleges in the United Arab Emirates, namely, Ajman University (AU) and Ras Al- Khaimah College of Dental Sciences (RAKCODS). It was conducted during the period from October 2019 to February 2020. An invitation to participate were sent to dental students on their clinical stage of practice only. The Ethics Committee of both universities approved the current cross-sectional study. Before the start of the study, the students were notified that their participation was voluntary and that refusing to participate would not affect their grades. Information that could identify the students was not collected.

This study used an online self-administrated questionnaire in English language on Microsoft Forms software. The questionnaire used was a modified version of the Standardized Nordic Questionnaire [12], and it was previously validated by other researchers [13, 14]. The questionnaire was composed of four parts. The first part comprised of questions related to socio-demographic data, specifically, gender. The second part included questions on the risk factors, such as history of physical trauma, family history of musculoskeletal disorders, exercise, caffeine consumption, the period of time spent in the clinic each day and smoking. The third part included questions linked to the occurrence of pain in the neck, shoulders, and low-back during the previous week and the previous twelve months. Furthermore, the participants were enquired about their height, weight, and number of hours spent studying or using computers to assess their physical activity level (Appendix 1).

Before the commencement of the study the questionnaire was pilot tested by the authors among 18 students to ensure understandabilities of the questions. The pre-testing showed a very good understanding of the provided questions. An online self – administered questionnaire was sent to all dental students in their clinical stage of practice through their batch email addresses and a cover letter to explain the purpose of the study were included. Confidentiality and anonymity of their responses were assured. The average time taken to complete the survey was nearly 5 minutes. Data were entered and analyzed using Statistical Package for Social Sciences version 22.0 (SPSS Inc., Chicago, IL, USA). Descriptive analysis was performed to obtain the frequencies, means, standard deviations and medians. For continuous variables the normality test was conducted. The frequency and percentage of MSP in the past 7 days and in the past year for the neck, shoulder and back was obtained. The chi- square test was conducted to get the crude odds ratio and 95% confidence interval.

## Results

In this cross-sectional study, questionnaires were completed by two hundred and two dental students (out of 368) from both colleges (AU and RAKCODS), with a total response rate of 55%. Missing data was excluded from the analysis. The majority of the students were female (75.2%). Most of the students (80.2%) had no history of trauma to the neck, shoulders, or lower back. Similarly, more than three-quarter of the participants (84.7%) had no family history of MSD. Approximately two-thirds (64.9%) of the students exercised regularly, and consumption of coffee was common among more than three-quarters of the participants (76.7%). The majority of the students (73.3%) spent 4 h per day in the clinics, and one-fifth (18.8%) of the participants used tobacco. The mean height (±SD) and weight (±SD) were 165 cm (±8.8) and 66.5 kg (±14.4), respectively. The sociodemographic data are shown in Table 1.

The prevalence of MSP in at least one body site was 48.5% in the past week and 68.3% in the previous year. Low-back pain was most common, with the prevalence rates of 38.6 and 61.4% in the previous week and previous year respectively. The prevalence of neck pain was 28.7% in the past week and 52.5% in the previous year. Shoulder pain was the least experienced in the previous week, and in the previous year (23.3, 44.1% respectively) as illustrated in Table 2.

The overall prevalence of MSP, regardless of the time of occurrence was not significantly associate with gender (OR 1.19, 95% CI 0.62–2.29, *P* = 0.608). A history of trauma was significantly associated with any MSP at any time. A family history of trauma was not significantly associated with MSP (*P* = 0.212). There was a significant association between exercise and the prevalence of MSP (*P* = 0.001), those who exercise regularly experienced less MSP. The consumption of caffeine was not significantly associated with MSP (*P* = 0.932). The duration of time spent in the clinics each day was significantly associated with MSP (*P* = 0.000). Similarly, body mass index (BMI) was significantly associated with MSP (*P* = 0.010). There was no significant association between smoking (*P* = 0.311), the duration of studying (*P* = 0.451) or the duration of computer use (*P* = 0.420) as illustrated in Table 3.

## Discussion

This cross-sectional study examined the prevalence of and factors associated with MSDs among dental students, by means of an online questionnaire. Similar studies have been conducted in Malaysia [9] and Czech Republic [10]; however, the characteristics of Ajman University students are very distinctive because Ajman University is the most diverse institute of higher education in the region; it is home to students from more than 70 nationalities, coming with totally different cultural backgrounds, which might have an impact on their musculoskeletal symptoms.

To our knowledge, this is the first study to report on MSDs among dental students in UAE. The main limitation of this type of research is that participants’ answers may not reflect their actual actions; which may possibly introduce some level of response bias. Nevertheless, this bias was limited as much as possible by utilizing a survey proven to be a valid and reliable tool for measuring the prevalence of MSDs [15]. Additionally, only two of the four dental colleges in the UAE were included, which might have an impact on the generalizability of the findings. In this study, the dental students were asked to note the occurrence of musculoskeletal pain over the past 12 months and the previous 7 days. The pain intensity and frequency were not evaluated in this study.

The results of this study showed that 68.3% of the students reported symptoms of musculoskeletal disorders on the last year. This percentage is comparatively lower than that reported studies reported by Khan and Chew [9], and Rabiei and colleagues [16]; and higher than other studies reported by Marshall and his team [17], and Ahmadi Motemayel and her team [18]. A similar percentage was reported by Finsen and co-workers [19], and Al-Ali and Hashim [11] who found that the prevalence of musculoskeletal pain in the past 12 months was 68.9 and 68.0% respectively. In the current study, only dental students in their clinical stage of practice were recruited, which might explain the high prevalence of MSP among them due to the work posture more common among clinical-year students.

Low-back pain occurrence in the past 12 months was the highest, experienced by more than half of the students, whereas the incidence of pain in this region in the past 7 days was reported by more than one-third. The high level of low-back pain might be due to holding a static load for long period of time, which in return might create more strain on dentists’ spine while delivering the dental care [2]. This clearly indicates that there is inadequate support of the lumbar region when students rest on the dental stool, and in fact, ergonomic principles are underestimated. Therefore, adequate support of the lumbar region is very critical since it helps preserve the lumbar curve. As a result, muscle activity, disc pressure, and back and leg pain will be decreased [20].

Neck pain in the past 12 months had a prevalence of 52.5%; the neck was the second most common reported anatomical region affected. This result supports the findings of similar previous studies conducted in other countries, such as the findings of Al Wazzan and co-workers [21], and Varmazyar and co-workers [22], who suggested that pain was mainly localized in the back and in the neck. The substantial prevalence of pain in the neck in this study could be associated with repetitive work performed with a flexed neck and elevated/abducted arms [23]. Even with their very short clinical experience, the high prevalence among undergraduate students suggests that the progression and deterioration of MSDs starts from the very beginning of the dentists’ career life [20]. Therefore, the importance of practicing dentistry while maintaining a proper working posture cannot be underestimated, as it greatly reduces the risk of work-related musculoskeletal disorders.

According to this research, it is crucial to implement ergonomic rules in the clinics to adapt the students to the correct working position. Certain precautions should be taken into consideration to reduce the fatigue and probability of developing MSDs [9]. If not taken seriously, students will develop serious symptoms over time, which will negatively interfere with their careers.

In this study, there was no statistically significant relation between gender, family history with trauma and MSP, which contradicts the findings of a previous study conducted by Waersted and co-workers [24]. The significant association between history of trauma and MSP in the current study is understandable. Individuals who experienced a traumatic injury to the neck, shoulder and lower back were at risk of developing MSP [25]. The insignificant association between other variables, such as coffee consumption, exposure to smoking, and the use of computers, is in agreement with findings reported by Algarni and co-workers [25].

Self-awareness and benefits of regular exercise are a necessity. Approximately one-fifth of the participants do not exercise at all. This behaviour might be a direct cause of MSP being more prevalent among clinical-years students in this study. This finding was also supported by Hashim and Al-Ali [26] who suggested that most dentists did not find as much time as they would like for exercise. According to a study performed by de Carvalho and co-workers [27], regular exercise can help prevent work- related musculoskeletal disorders, and those who participate in any kind of sports activity experience less severe symptoms than those who are not physically active. Regular exercise may provide dental students with the required break from their heavy workload, which will recharge and strengthen their bodies. It will also provide mental relaxation from high psychosocial demands of the job [23]. These effects probably interact to contribute to a better health status and, in return, will decrease the risk of MSP [28].

According to this study, the time spent in the clinic was significantly related to experiencing musculoskeletal problems, which is in accordance with previous studies conducted among dental professionals [3, 21, 29, 30]. This might be attributed to the fact that dentistry demands a high level of precision, and it is often preformed with the arms unsupported and the cervical spine rotated and flexed forward [31]. Additionally, holding static load for long durations may cause symptoms associated with the mask system [32]. In a study carried out by Melis and co-workers [33], the data suggested that MSDs are prevalent in dental students in Italy as soon as they start their clinical practice in the clinics. For this reason, it is highly recommended to educate students about ergonomics throughout their clinical training periods to avoid the complications of MSDs [33].

The results of this study suggest a significant relationship between BMI and the prevalence of MSP, which is consistent with the findings of Ahmadi Motemayel and co-workers [18], and Betterworth and co-workers [34]. It is worth noting that work-related musculoskeletal disorders not only have negative psychological and social outcomes but can also become severe to the point that they have a direct effect on work capacity and might even lead to early job retirement [27]. As a result, the evaluation of dental students’ knowledge about work- related musculoskeletal disorders is crucial.

## Conclusion

The present study indicates that the prevalence of MSP in the previous year is high among undergraduate dental students, particularly those with a history of trauma, less physical activity, long clinical sessions hours and a higher BMI. This problem is of particular concern; therefore, careful attention by dental colleges is necessary to increase students’ awareness of MSDs; exercise and weight reduction should receive special attention. Further continuing education and investigation of appropriate interventions to reduce this problem is needed.

## Supplementary Information

**Additional file 1: Table S1.** Demographic characteristics of participants (*N* = 202). **Table S2.** Prevalence of MSP during the past week and past 12 months (N = 202). **Table S3.** Factors associated with MSP in at least one body site at any time.

**Additional file 2.** Questionnaire (The Musculoskeletal Disorders Among Dental Students in the UAE).

## Data Availability

The datasets used and/or analyzed during the current study are available from the corresponding author on reasonable request.

## Abbreviations

AU: Ajman University
BMI: Body Mass Index
MSD: Musculoskeletal Disorder
MSP: Musculoskeletal Pain
*P*: Probability value
RAKCODS: Ras Al- Khaimah College of Dental Sciences
SD: Standard Deviation
SPSS: Statistical Product and Service Solutions
UAE: United Arab Emirates
