# Supplementary material for: Prevalence of postural musculoskeletal symptoms among dental students in United Arab Emirates
Source: BMC Musculoskelet Disord. 2021 Jan 6;22:30. doi: 10.1186/s12891-020-03887-x (PMC7788996; doi:10.1186/s12891-020-03887-x)
Supplement: Supplementary file 1 — Additional file 1: Table S1. Demographic characteristics of participants (N = 202). Table S2. Prevalence of MSP during the past week and past 12 months (N = 202). Table S3. Factors associated with MSP in at least one body site at any time. [file 12891_2020_3887_MOESM1_ESM.docx]

Table-1 Demographic characteristics of participants (N = 202).

| **Variables** | **N** | **%** |  |
| --- | --- | --- | --- |
| Gender |  |  |  |
| Male | 50 | 24.8 |  |
| Female | 152 | 75.2 |  |
| History of trauma in the neck, shoulder, lower-back | |  |  |
| Yes | 40 | 19.8 |  |
| No | 162 | 80.2 |  |
| Family History of MSD |  |  |  |
| Yes | 39 | 19.3 |  |
| No | 163 | 80.7 |  |
| Exercise |  |  |  |
| Regular | 131 | 64.9 |  |
| Occasional | 34 | 16.8 |  |
| Not at all | 37 | 18.3 |  |
| Coffee Consumption |  |  |  |
| < 3 cups / week | 65 | 32.1 |  |
| > 3 cups / week | 90 | 44.6 |  |
| Not at all | 47 | 23.3 |  |
| Duration of clinical session / day |  |  |  |
| 2 hours | 18 | 8.9 |  |
| 4 hours | 148 | 73.3 |  |
| 8 hours | 36 | 17.8 |  |
| Smoking |  |  |  |
| Yes | 38 | 18.8 |  |
| No | 164 | 81.2 |  |
|  |  |  |  |
|  | **Mean (SD)** | |  |
| Height | 165 | (8.8) |  |
| Weight | 66.5 | (14.4) |  |
| Hours of computer use / day | 3.7 | (2.1) |  |
| Hours of study / day | 3.3 | (2.0) |  |
|  |  |  |  |

Table-2 Prevalence of MSP during the past week and past 12 months (N = 202).

| **Body site** | **Prevalence of MSP  during the past week** | | **Prevalence of MSP  during the past 12 months** | |
| --- | --- | --- | --- | --- |
|  | **N** | **%** | **N** | **%** |
| Neck pain |  |  |  |  |
| Yes | 58 | 28.7 | 106 | 52.5 |
| No | 144 | 71.3 | 96 | 47.5 |
| Shoulder pain |  |  |  |  |
| Yes | 47 | 23.3 | 89 | 44.1 |
| No | 155 | 76.7 | 113 | 55.9 |
| Low-back pain |  |  |  |  |
| Yes | 78 | 38.6 | 124 | 61.4 |
| No | 119 | 58.9 | 76 | 37.6 |
| Over all (at least one site) | 98 | 48.5 | 138 | 68.3 |
|  |  |  |  |  |

Table-3 Factors associated with MSP in at least one body site at any time.

| **Variables** | **Yes** | | **(%)** | | **No** | | **(%)** | | **OR (95% CI)** | | | **P-value** | |  |
| --- | --- | --- | --- | --- | --- | --- | --- | --- | --- | --- | --- | --- | --- | --- |
| Gender |  | |  | |  | |  | |  | | |  | |  |
| Male | 31 | | (62.0) | | 19 | | (38.0) | |  | | |  | |  |
| Female | 88 | | (57.9) | | 64 | | (42.1) | | 1.19 (0.62-2.29) | | | 0.608 | |  |
| History of trauma in the neck, shoulder, lower-back | | | | |  | |  | |  | | |  | |  |
| Yes | 27 | | (67.5) | | 13 | | (32.5) | |  | | |  | |  |
| No | 72 | | (44.4) | | 90 | | (55.6) | | 2.60 (1.25-5.39) | | | 0.009 | |  |
| Family History of MSD |  | |  | |  | |  | |  | | |  | |  |
| Yes | 23 | | (59.0) | | 16 | | (41.0) | |  | | |  | |  |
| No | 78 | | (47.9) | | 85 | | (52.1) | | 1.57 (0.77-3.18) | | | 0.212 | |  |
| Exercise |  | |  | |  | |  | |  | | |  | |  |
| Regular | 84 | | (64.1) | | 47 | | (35.9) | |  | | |  | |  |
| Occasional | 15 | | (44.1) | | 19 | | (55.9) | |  | | |  | |  |
| Not at all | 12 | | (32.4) | | 25 | | (67.6) | |  | | | 0.001 | |  |
| Coffee Consumption |  | |  | |  | |  | |  | | |  | |  |
| < 3 cups / week | 34 | | (52.3) | | 31 | | (47.7) | |  | | |  | |  |
| > 3 cups / week | 45 | | (50.0) | | 45 | | (50.0) | |  | | |  | |  |
| Not at all | 23 | | (48.9) | | 24 | | (51.1) | |  | | | 0.932 | |  |
| Duration of clinical session / day | |  | |  | |  | |  | |  | | |  | |
| 2 hours | 6 | | (33.3) | | 12 | | (66.7) | |  | | |  | |  |
| 4 hours | 112 | | (75.7) | | 36 | | (24.3) | |  | | |  | |  |
| 8 hours | 32 | | (88.9) | | 4 | | (11.1) | |  | | | 0.000 | |  |
| Smoking |  | |  | |  | |  | |  | | |  | |  |
| Yes | 22 | | (57.9) | | 16 | | (42.1) | |  | | |  | |  |
| No | 80 | | (48.8) | | 84 | | (51.2) | | 1.44 (0.71-2.95) | | | 0.311 | |  |
|  |  | |  | |  | |  | |  | | |  | |  |
| **Yes (Median, IQR) No (Median, IQR)** | | | | | | | | | | | |  | |  |
| Body Mass Index | 126 (24.3, 4.9) | | | | 76 (23.0, 5.5) | | | | | |  | 0.010 | |  |
| Hours of computer use / day | 126 (3.0, 3.0) | | | | 76 (4.0, 3.0) | | | | | |  | 0.420 | |  |
| Hours of study / day | 126 (3.0, 3.0) | | | | 76 (3.0, 2.0) | | | | | |  | 0.451 | |  |
|  |  | |  | |  | |  | |  | | |  | |  |
