# Supplementary material for: Prevalence of postural musculoskeletal symptoms among dental students in United Arab Emirates
Source: BMC Musculoskelet Disord. 2021 Jan 6;22:30. doi: 10.1186/s12891-020-03887-x (PMC7788996; doi:10.1186/s12891-020-03887-x)
Supplement: Supplementary file 2 — Additional file 2. Questionnaire (The Musculoskeletal Disorders Among Dental Students in the UAE). [file 12891_2020_3887_MOESM2_ESM.docx]

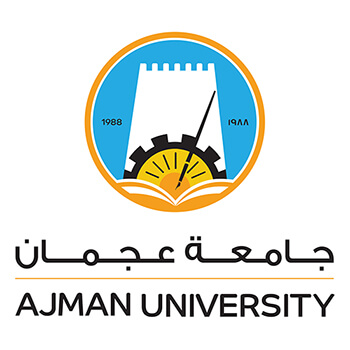


**The Musculoskeletal Disorders Among Dental Students in the UAE**

**Questionnaire**

*Kindly circle the most appropriate answer*

Gender: Male Female

Academic year: ---------------------

Do you have any history of trauma in the neck, shoulder, lower back? Yes No

Do you have any family history of musculoskeletal disorder? Yes No

Do you Exercise? Regularly Occasionally Not at all

Do you drink Coffee? < 3 cups/week >3cups/week Not at all

Do you smoke? Yes No

What is the average duration of your clinical session/day?

2 hours 4 hours 8 hours

How many hours per day do you use computer? _________

How many hours per day do you study? _________

What is your weight? _________

What is your height? _________

**Have you experienced any of the following?**

Neck pain during the past week Yes No

Neck pain during the past 12 months Yes No

Shoulder pain during the past week Yes No

Shoulder pain during the past 12 months Yes No

Lower back pain during the past week Yes No

Lower back pain during the past 12 months Yes No
